# Reliability and validity of 30-15 intermittent fitness test for cardiorespiratory fitness assessment among infantry members of Slovenian armed forces: A study protocol

**DOI:** 10.3389/fpsyg.2022.894186

**Published:** 2022-07-22

**Authors:** Armin H. Paravlic, Bostjan Simunic, Rado Pisot, Samo Rauter, Janez Vodicar

**Affiliations:** ^1^Faculty of Sport, University of Ljubljana, Ljubljana, Slovenia; ^2^Institute for Kinesiology Research, Science and Research Centre, Koper, Slovenia; ^3^Faculty of Sports Studies, Masaryk University, Brno, Czechia

**Keywords:** visual attention, 30-15 intermittent fitness test, running endurance, validation, military personal

## Abstract

**Introduction:**

Cardiorespiratory fitness (CRF) testing is routinely performed by most armed and tactical forces around the world as part of their recruitment process for new members or simply as an annual examination of their personnel. A 2-mile run (2_MR_) test is among the most commonly used. However, as it is performed outdoors, weather, climate, and terrain can influence the results and often limit the maximum performance. Also, this test has been shown to be difficult for individuals because the pacing strategy is self-paced. As such, it does not reflect the real situation on the battlefield, where most activities are externally driven by the environment and the enemy. Therefore, we believe that the 30-15 Intermittent Fitness Test (30-15_IFT_) may be a suitable tool for measuring CRF and battle readiness of military personnel. Moreover, given the importance of visual attention to military personnel we aim to investigate the differences in visual attention between better and less physically prepared infantry members and its corresponding response to maximal endurance running test.

**Methods and analysis:**

This randomized cross-over study using a within-subjects test-retest design will enroll 32 infantry members of the Slovenian Armed Forces. To investigate the reliability and validity of the 30-15_IFT_ test, an incremental treadmill test (TR), a 2_MR_ test, and two identical 30-15_IFT_ will be performed in randomized order. Additionally, participants will be subsequently divided into two groups based on their score on the Army Physical Fitness Test (APFT), whereas differences in visual attention will be assessed by using the d2 test. The primary analysis will assess differences in key physiological outcomes between the different CRF tests (TR vs. 2_MR_ vs. 30-15_IFT_). In addition, the relative reliability of all dependent variables between two 30-15_IFT_ trials will be estimated by interclass correlation coefficient (ICC), while relationship between maximal oxygen uptake, heart rate and maximal running speed of 30-15_IFT_, TR and 2_MR_ will be assessed using Pearson’s correlation.

**Ethics and dissemination:**

Ethical approval was obtained from the National Medical Ethics Committee (reference number: 0120-495/2021/6). The results of the proposed study will be disseminated through publication in peer-reviewed journals.

**Clinical Trial Registration:**

[www.ClinicalTrials.gov], identifier [NCT05218798].

## Strengths and limitations of this study

– This study will be the first to investigate the reliability and validity of the 30-15 Intermittent Fitness Test in military personnel.
– This study will be the first to compare three different protocols for assessing cardiorespiratory fitness assessment in military personnel.
– The study will provide the adopted formula for accurate estimation of maximal oxygen uptake (VO_2_max) for infantry members of the Slovenian Armed Forces (SAF) from the 30-15 Intermittent Fitness test.
– The study will provide information about differences in visual attention between better and less physically prepared members of military personnel and its corresponding response following maximal endurance running test. This will enable better understanding of the importance of physical fitness for cognitively demanding situations like those at the battlefield.
– A potential limitation of the study is that we will recruit greater number of male than female SAF members, as it is difficult to find an adequate number of female participants who meet the pre-specified inclusion criteria.

## Introduction

Everyday military tasks consist of various movement patterns such as walking, running, jumping, pushing, pulling, lifting, throwing, kicking, dragging, crawling, shooting a firearm, carrying a heavy backpack, and/or other equipment, all of which place a high level of stress on the soldier’s body and mind (Knapik and Whitfield, 2014). Therefore, in order to perform later tasks with ease and greater efficiency, soldiers must possess a high level of physical fitness (Knapik and Whitfield, 2014; Orr et al., 2021). Similar to other physically demanding occupations such as firefighting, and/or law enforcement, muscular strength, strength endurance, and cardiorespiratory fitness (CRF) have been shown to be one of the most important components of a soldier’s physical fitness profile (Herrador Colmenero et al., 2014; Dawes et al., 2021). To test these attributes, there are dozens of individual tests and test batteries, of which the Army Physical Fitness Test (APFT) is the most widely used test battery for military personnel due to its simplicity and ease of administration (Herrador Colmenero et al., 2014; Knapik and Whitfield, 2014). The APFT, which consists of sit-ups, push-ups and a 2-mile run (2_MR_), was introduced in 1980 for members of the U.S. Army. It is design to measure upper body and core muscles strength endurance and CRF (Herrador Colmenero et al., 2014; Knapik and Whitfield, 2014). While muscle strength and core muscle strength endurance are relatively easy to measure, assessing CRF in this special population appears to be more challenging due to the specifics of military operations, which are complex and often unpredictable (Aandstad et al., 2011; Knapik and Whitfield, 2014; Canino et al., 2018). CRF testing is routinely performed by most armed and tactical forces around the world as part of their recruitment process for new members or simply as an annual examination of their personnel (Aandstad et al., 2011; Knapik and Whitfield, 2014). Indeed, direct measurement of CRF during an incremental run on a treadmill (TR) is considered the “gold standard” for estimating maximal oxygen uptake (Vo2max) (Albouaini et al., 2007). However, because of its high cost, complex measurement procedures, and inability to measure more than one person at a time, this method is not routinely used to examine CRF in soldiers (Herrador Colmenero et al., 2014; Knapik and Whitfield, 2014). Therefore, in addition to the 2_MR_, several other field tests such as the 12-mile run, the 3,000-m run, the 2,400-m run, and the 1-mile run are used for CRF evaluation of military personnel (Herrador Colmenero et al., 2014; McGuire and Lockie, 2021). Later tests are usually conducted outdoors. Therefore, weather, climate, and terrain can influence the results and often limit the maximum performance. Although standardized, the most commonly used tests, such as the 2_MR_, have been shown to be difficult for individuals because the pacing strategy is internally controlled (Aandstad et al., 2011). As such, it does not reflect the real situation on the battlefield, where most activities are externally driven by the environment and the enemy. Running patterns on the battlefield are characterized by intermittent, high-intensity shuttle runs with constant changes of direction combined with forward and backward running and turning, while unexpected interruptions of running are common when soldiers are exposed to open fire from the enemy. In those situations, a high level of attention is necessary to support fast and right-decision making to save its’ own live and the lives of other soldiers and civilians. In addition, a high standard of strength and conditioning planning and programming required for military personnel cannot be met with such a test because the final result is expressed as the total time required to run a given distance without any other information needed for training prescription and necessary optimization. Therefore, a new test should be introduced for CRF testing of soldiers that takes into account all of the above, including the specifics of the environment and running patterns similar to those happening on the battlefield (Newell, 1989). Over the past two decades, an intermittent shuttle run testing with 30-15 Intermittent Fitness Test (30-15_IFT_) has been successfully implemented for CRF testing in various populations (Buchheit, 2010; Rabbani and Buchheit, 2015). 30-15_IFT_ is an incremental test consisting of 30-s shuttle runs interspersed with 15-s active recovery periods. At the beginning of the test, a running speed of 8 km/h is set for the first 30-s run and increased by 0.5 km/h in each 30-s phase thereafter. Subjects must run back and forth between two lines 40 m apart at the specified pace determined by a pre-recorded beep. The speed of the last successfully completed stage was recorded as the test result and represents the maximum running speed (MRS) during 30-15_IFT_ (MRS_*IFT*_) (Buchheit, 2008). It has also been shown to reliably predict the VO2max values, suggesting that it can be used for CRF assessment as well as individual training prescription (Buchheit, 2008, 2010). Considering all of the above, we believe that the 30-15_IFT_ may be a suitable tool for measuring CRF of military personnel.

To date, no study has examined the reliability and validity of the 30-15_IFT_ compared to a standard continuous incremental running test and/or a 2_MR_ in infantry members of the Slovenian Armed Forces (SAF). From a practical perspective, it would be of great interest to SAF personnel to provide their strength and conditioning coaches with a valuable measure of CRF to determine and monitor the readiness of SAF infantry members. In addition, we aimed to investigate differences in visual attention between better and less physically prepared infantry members and its corresponding response to maximal endurance running test. Therefore, we hypothesized that the 30-15_IFT_ will prove to be a highly reliable and valid measure of CRF while other parameters such as maximal heart rate (HR_*max*_) and maximal running speed (MRS_*IFT*_) will be valuable indicators of intermittent fitness data for prescribing, monitoring, and optimizing high-intensity interval training in this population. Moreover, it is expected that when compared to less prepared infantry members, a better prepared infantry member will have fewer decline of visual attention following maximal endurance running test.

## Materials and methods

### Study design

The proposed study is a part of a greater project entitled “Enhancement of Physical and Combat Preparedness of Slovenian Armed Force Members; “(original language, Slo: “Dvig telesne in bojne pripravljenosti pripadnikov Slovenske vojske”), which was funded by the Slovenian Research Agency (ARRS) and Slovenian Ministry of Defense (project No. V5-2106; project manager: Assoc. Prof. Dr. Janez Vodičar; principal investigator: Assist. Prof. Dr. Armin Paravlic). This is a randomized cross-over study using a within-subjects test-retest design. To test the current hypothesis, an incremental treadmill test (TR), a 2_MR_ test, and two identical 30-15_IFT_ will be performed in the population of SAF soldiers. For a better understanding of the following text, the term participant is used instead of soldier. The present study will be conducted over a maximum period of 2 weeks per participant. Participants will be instructed to visit the laboratory four times at least 72 h apart. At the first visit, they will be familiarized with the experimental procedures and asked to sign a written informed consent form to participate in the study. Then, participants will be randomly assigned to four experimental conditions (i.e., TR, 2_MR_ and two 30-15_IFT_ running tests).

Additionally, participants will be subsequently divided into two groups based on their score on the APFT (score ranging from 1 to 5). Thus, the higher-scoring group (HSG) is defined as an APFT score of ≥ than 4; and the lower-scoring group (LSG) is defined as an APFT score of ≤ than 2.

### Participants

Thirty-two SAF infantry members (males, *N* = 27) will be recruited for the proposed study. Inclusion criteria are: age 18–40 years, both male and female SAF infantry members who are performing regular military tasks on daily basis, with no history of injuries in the last 6 months prior to recruitment, have not reported any musculoskeletal pain, with no history of chronic musculoskeletal, metabolic, pulmonary, neurological and cardiovascular diseases. Participants will be instructed to avoid any strenuous physical activity for at least 3 days prior to the start of the first testing session and during the course of study, which will be monitored by their superiors. They will be requested to refrain from consuming ergogenic substances and caffeinated beverages prior to the tests. Before the initial assessment on each testing day, a brief meeting will be held to explain the study protocol in detail.

### Experimental protocol

All tests will be performed at the facilities at the Faculty of Sport (University of Ljubljana, Slovenia) between 8 a.m. and 13 p.m. The actual arrival of participants to the testing location will be pre-planned and coordinated with the commanding officers. Each testing group will have 6–8 members in total and they will be instructed to arrive at least 15 min before actual testing, that is 7:45 A.M. The testing procedures will be than explained in detailed and demonstrated by the well experienced faculty staff members. The timeline of testing procedures is explained in detail in Table 1.

**TABLE 1:** Timeline of study protocol.

Activity/Measurements	Time (min)
Body height, weight, and body composition	5
Test of visual attention (d2)	5–10
Supine rest (initial heart rate monitoring)	10–15
Warm-up routine—upright cycling 5 min (1 w pr kg of body weight, 70 rpm) Part I	15–20
Dynamic stretching Part II	20–24
Specific warm-up (running 8 km/h) Part III	24–30
Actual aerobic test (TR, 2_RM_ or 30-15_IFT_)	30–55
Supine rest (post-test heart rate monitoring)	55–60
Test of visual attention (d2)	60–65

TR, treadmill run test; 2_RM_, 2-mile running test; 30-15_IFT_, Intermittent Fitness Test; rpm, revolutions per minute.

### Measurements

#### Anthropometrics

Body mass and height will be measured using a stadiometer and scale anthropometer (GPM, Model 101, Zurich, Switzerland) to the nearest 0.1 cm, while body mass was assessed with multifrequency bioelectrical impedance (InBody 720: Biospace Co., Ltd., Seoul, South Korea) to the nearest of 0.01 kg. Additionally, fat mass, body fat percentage, skeletal muscle mass, fat free mass and total body water were calculated using manufacturer’s algorithm like elsewhere (Seino et al., 2015).

#### Attention assessment

The d2 test was used to determine the level of concentrated visual attention of participants (Bates and Lemay, 2004). It consists of 14 rows with 47 characters per line. These characters are the letters d or p, with a total of one to four dashes located above and/or below each letter. Participants will be instructed to scan each line and cross out only the characters containing the letter d with two dashes during 20 s. After completion of the d_2_ test, two variables will be calculated—concentration performance (CP) and total number of total errors (TE) made by the soldiers. CP is calculated as the number of correctly marked d_2_-symbols minus the number of incorrectly marked symbols (symbols that are not d_2_-symbols). The TE will be assessed as the number of errors made by participants by failing to correctly identify a d_2_-symbol plus the number of errors made by incorrectly marking symbols that were not d_2_- symbols.

#### Maximal aerobic capacity testing equipment

A K5 a portable gas analyzer (COSMED, Italy) will be used to obtain physiological parameters. The device provides reliable values for oxygen uptake volume (VO_2_), carbon dioxide production volume (VCO_2_), and pulmonary ventilation (VE) in a breath-by-breath manner. In addition, capillary blood samples from the earlobe will be collected for all tests (before, immediately after, 3 min after and 5 min after) and the samples will be analyzed for blood lactate concentration [(LA^–^)] using a Biosen C-line analyzer (EKF Diagnostics, Germany).

Simultaneously, a heart rate will be measured by Garmin Edge 830 Pack heart rate monitoring belt (Kansas, United States). The data will be recorded in 5-s interval and automatically analyzed by using the original Polar software.

#### Incremental treadmill test

After 5 min of baseline measurements, while standing on the treadmill (HP Cosmos, Germany), the participants will warm up at 8 km/h run and constant gradient of 1% inclination (Jones and Doust, 1996). Then, they will execute the actual test by running until volitional exhaustion where the running speed will be increased progressively by 2 km/h per minute. The achievement of VO_2max_ was identified as the plateauing of VO_2_ (<2.1 ml/kg/min decrease) despite an increase in workload (Poole and Richardson, 1997). If the above-stated criterion was not fulfilled, the participants will be asked to perform a further constant-speed test equal or higher than the highest speed achieved at the end of the incremental test, as recommended (Rossiter et al., 2006). Throughout the test, respiratory gases will be continuously measured breath-by-breath and reduced to 10-s averages (Borszcz et al., 2018). MRS will be determined as the minimal running velocity that elicited VO_2_max over a period of 30 s.

#### 2-mile run test

The 2_MR_ will be used as a continuous field test to assess CRF. It will be performed on a 400-m synthetic athletic track with the supervision of the research team. Participants will be required to complete the 2-mile run course without any physical help in the shortest time possible. At the start, all participants will line up behind the starting line. On the command “go,” the clock will start. They will begin running at their own pace. Although walking is authorized, it is strongly discouraged. Immediately after competition of the test, the time achieved will be reported to the nearest to 0.01 s and used for further analysis. The heart rate at the end of the 2-mile test will be considered the maximal heart rate achieved in the test. Additionally, the perceived effort of the trial will be recorded at the end of the test using the modified Borg visual scale (Borg, 1982).

#### 30-15 Intermittent fitness test

30-15_IFT_ test will be used as the field-based assessment tool to measure CRF, as previously recommended (Buchheit, 2008; Mohorič et al., 2021). This intermittent, incremental test consists of 30-s shuttle runs interspersed with 15-s active recovery periods. Running speed was set at 8 km/h for the first 30-s run and increased by 0.5 km/h in each 30-s phase thereafter. Participants will be required to run back and forth between two lines 40 m apart at the predetermined pace determined by a prerecorded beep. The prerecorded beep allowed participants to adjust their running speed when they entered a 3-m zone in the middle and at each end of the test field. During the 15-s recovery period, participants will walk to the nearest line (either in the middle or at the end of the running area, depending on where their previous run had ended); from this line, they began the next running phase. Participants will be instructed to complete as many stages as possible. The test ended when the participant could no longer maintain the required running speed or failed to reach the 3-m zone three consecutive times in time before the sound signal. The speed of the last successfully completed stage was recorded as the test result, i.e., the MRS during 30-15_IFT_ (MRS_IFT_) (Buchheit, 2008). The VO2max was calculated by following formula (ref):


$$V\,O_{2}\,m\,a\,x\,I\,F\,T\,\left(\frac{m\,l}{\frac{m\,i\,n}{k\,g}}\right)=28.3-2.15\,S-0.741\,A-0.0357\,B\,M+0.058\,A\times M\,R\,S_{I\,F\,T}+1.03\,M\,R\,S_{I\,F\,T}$$

Whereas (G) stands for sex, (A) for age and (BM) for subjects’ body mass, respectively.

#### Rating of perceived exertion

Before and after each session, the “Rating of Perceived Exertion” (RPE) scale was used to estimate the participants’ perceived effort. It ranged between 0/“no perceived effort” (i.e., rest) and 10/“maximal perceived effort” (i.e., the most stressful exercise ever performed) (Borg, 1982).

### Sample size calculation and statistical analysis

#### Sample size

In the conceptualization phase of the study, we conducted an *a priori* power analysis for the estimated interclass correlation coefficient (ICC) as previously recommended (Bujang and Baharum, 2017). Based on previous studies with similar aim we expected to find high to nearly perfect ICC for the reliability of 30-15_IFT_ test (Buchheit, 2008; Buchheit et al., 2011). Therefore, with power of 0.90, two-tiled α = 0.05, and four observations per subject a minimum sample size of 6 subjects showed to be sufficient to detect a value of ≥ 0.80 for the ICC.

#### Statistical analysis

All data will be presented as mean ± SD with 90% confidence interval limits (90% CI). All statistical analysis will be conducted using the SPSS statistical software (version 27.0, IBM Inc., Chicago, United States). Descriptive statistics will be used to summarize demographic characteristics of participants and outcomes. Normality od the data will be confirmed by using the visual inspection of histogram and Q-Q plot and analytically by Shapiro-Wilk test, while the homogeneity of variances of normally distributed variables will be tested by using Levene’s test.

Differences in key physiological outcomes between the different CRF tests (TR vs. 2_MR_ vs. 30-15_IFT_) will be assessed by One-Way ANOVA analysis. The relative reliability of all dependent variables between two 30-15_IFT_ trials will be estimated using the ICC, two-way random effects model (consistency type). ICC values will be classified as very high if > 0.90, high if between 0.70 and 0.89, and moderate if between 0.50 and 0.69. The following criteria will be used to declare good reliability: CV < 5% and ICC > 0.69 (Buchheit et al., 2011). In addition, the standard error of the estimate (SEM) and the coefficient of variation (CV) will be calculated as measures of absolute reliability, indicating the within-subject variation, as previously proposed (Hopkins, 2000). To further address the reliability of the data, bias and random error will be calculated, followed by the minimal detectable change (MDC). To test the usefulness of the IFT, the spreadsheet provided by Hopkins will be used (Hopkins). Usefulness will be determined by comparing the smallest worthwhile change (SWC) with the typical error of measurement (TE) and will be interpreted as follows: “Marginal” (TE > SWC), “OK” (TE = SWC) and “Good” (TE < SWC) (Hopkins, 2007).

Relationship between VO_2max_, HR_peak_ and end running speed of 30-15_IFT_, TR, and 2_MR_ will be assessed using Pearson’s correlation (r). Also, the relationship between VO_2max_ obtained from TR, 2_MR_, and 30-15_IFT_ will be investigated. The following thresholds of the correlation coefficient will be used to assess the magnitude of the relationships analyzed: weak ≤ 0.35; 0.36 ≤ moderate < 0.67; 0.68 ≤ high < 1 (Taylor, 1990).

In addition, to determine performance differences between two groups of SAF members, a repeated-measures General Linear Model will be used for main physiological parameters estimated from different tests (TR vs. 2_MR_ vs. 30-15_IFT_) as within-subject factor, whereas groups (HSG vs. LSG) will be used as between-subject factors. Partial eta squared (η^2^) values of 0.01, 0.06, and 0.14 rated difference as small, moderate and high, respectively (Cohen, 1988). Statistical significance for all analysis conducted will be accepted at *p* ≤ 0.05.

### Ethics and dissemination

In designing the proposed study, all ethical issues were thoroughly considered and all necessary National Medical Ethics Committee approvals were obtained before this protocol was submitted (reference number: 0120-495/2021/6). Sample size was appropriately estimated and verified after pilot testing to ensure valid statistical analysis and testing of the main hypothesis. Informed consent will be obtained from all study participants. The participant information sheet provides details about the study, the implications and limitations of the study protocols, and any known side effects or risks associated with participation in the study. It also states that the participant is free to withdraw from the study without impact on their future status and without having to provide the reason for their withdrawal. Protection of personal data is ensured by encrypting names so that no information about subjects is revealed during analysis and/or publication. The experimental methodology and the measuring instruments used have been tested several times by us and other researchers and are absolutely non-invasive. All instruments used have an appropriate certificate for human use with all necessary safety features. The results of this study will be presented at various scientific meetings, conferences, mentorships, etc. Due to the amount of work we will be doing, we also plan to publish at least two master’s theses and probably one doctoral thesis in this field, as well as two high quality publications in internationally peer-reviewed journals.

### Patient and public involvement

Patients and/or the public were not involved in the design and/or conduct, and/or reporting of this study. Participants of the proposed study will be invited to share their experiences of their involvement in our study protocols with other members of the Slovenian Armed Forces. The authors will gather participants’ experience with the proposed study protocols to structure feasible evaluation protocols for the second part of the project.

## Data availability statement

The original contributions presented in this study are included in the article/supplementary material, further inquiries can be directed to the corresponding author/s.

## Ethics statement

The studies involving human participants were reviewed and approved by the National Medical Ethics Committee of the Republic of Slovenia. The patients/participants will provide their written informed consent to participate in this study.

## Author contributions

AP, JV, RP, and BS: conceptualization. AP: methodology, software, and writing—original draft preparation. AP and SR: validation, investigation, formal analysis, and data curation. JV and AP: resources, writing—review and editing, and funding acquisition. AP, JV, and SR: project administration. All authors contributed to the article and approved the submitted version.

## Conflict of interest

The authors declare that the research was conducted in the absence of any commercial or financial relationships that could be construed as a potential conflict of interest.

## Publisher’s note

All claims expressed in this article are solely those of the authors and do not necessarily represent those of their affiliated organizations, or those of the publisher, the editors and the reviewers. Any product that may be evaluated in this article, or claim that may be made by its manufacturer, is not guaranteed or endorsed by the publisher.
